# Colloidal Gold Dietary Supplements as Nanomaterials: Physicochemical Evaluation, Estimated Oral Exposure, and Preliminary Biological Assessment

**DOI:** 10.3390/ijms27135872

**Published:** 2026-06-29

**Authors:** Oana Catalina Bute, Anca Irina Gheboianu, Andreea Neacsu, Carmen Curutiu, Ionela Avram, Lia Mara Ditu

**Affiliations:** 1 Faculty of Sciences and Arts, Valahia University of Târgoviște, 13 Sinaia Alley, 130004 Târgoviște, Romania; oana.bute@valahia.ro; 2 Institute of Multidisciplinary Research for Science and Technology, Valahia University of Târgoviște, 13 Sinaia Alley, 130004 Târgoviște, Romania; 3 Institute of Physical Chemistry “Ilie Murgulescu”, Romanian Academy, Splaiul Independentei 202, 060021 Bucharest, Romania; addneacsu@icf.ro; 4 Microbiology Department, Faculty of Biology, University of Bucharest, Intr. Portocalelor 1–3, 060101 Bucharest, Romania; lia-mara.ditu@bio.unibuc.ro; 5 Genetic Department, Faculty of Biology, University of Bucharest, Intr. Portocalelor 1–3, 060101 Bucharest, Romania; ionela.sarbu@bio.unibuc.ro

**Keywords:** gold nanoparticles, colloidal gold, dietary supplements, engineered nanomaterials, oral exposure, cytotoxicity, lactic acid bacteria

## Abstract

Colloidal gold dietary supplements intended for oral consumption are increasingly marketed as nano-enabled products, yet their physicochemical characteristics and biological effects remain insufficiently documented. In this study, commercially available colloidal gold supplements produced and marketed in Romania (30, 55, and 110 mg/L) were investigated to determine their classification as nanomaterials and to assess their preliminary biological effects in the context of oral exposure. Transmission electron microscopy revealed a narrow particle size distribution (4–11 nm), while SAED and EDX confirmed the presence of metallic gold nanoparticles. UV-VIS spectroscopy showed the characteristic surface plasmon resonance, supported by comparison with citrate-stabilized reference AuNPs (5–20 nm). DLS and zeta potential measurements indicated stable electrostatically stabilized colloids. According to the current EU definition, the number-based size distribution supports classification as nanomaterials. Manufacturer-recommended daily intakes were compared with doses reported in the literature using HED conversion to contextualize oral exposure. In vitro assays showed no pronounced acute cytotoxic or antitumoral effects on HCT-8 cells and no inhibitory effects on selected LAB. However, increased cytotoxicity was observed in HEK293 cells exposed to the dietary supplement formulation compared with the corresponding standard AuNP formulation. These results underscore the importance of considering cell-specific responses when evaluating the safety of nano-enabled dietary supplements and support the need for long-term toxicological studies.

## 1. Introduction

Nanomaterials exhibit physicochemical properties that differ substantially from those of their bulk counterparts due to their small size, high surface-area-to-volume ratio, and size-dependent interactions with biological systems [1,2,3,4]. Regulatory frameworks recognize these differences through specific definitions of nanomaterials, including those provided by the European Commission, ISO, OECD, and the EU Novel Food Regulation [5,6,7,8,9]. These definitions reflect the need for dedicated characterization and safety assessment strategies when human exposure to nanoscale materials is expected [10]. Among engineered nanomaterials, gold nanoparticles (AuNPs) have attracted considerable attention because of their chemical stability, relative biocompatibility, and unique optical properties associated with localized surface plasmon resonance (LSPR) [11,12,13,14]. These characteristics have enabled their use in biomedical imaging, biosensing, targeted drug delivery, photothermal therapy, and other cancer-related applications [14,15,16,17,18,19]. Several studies have reported antiproliferative and pro-apoptotic effects of AuNPs in malign cell lines [20,21,22,23,24,25,26], whereas others have demonstrated limited biological activity, indicating that their antitumoral potential depends strongly on nanoparticle size, shape, surface chemistry, concentration, and biological model employed [27,28,29].

More recently, colloidal gold has been commercialized as a dietary supplement despite the absence of formal authorization for oral ingestion within the European Union [30,31]. Manufacturers frequently attribute benefits such as stimulation of the reticuloendothelial system, support of cellular growth and regeneration, regulation of digestive processes, antioxidant activity, and potential antitumoral effects to these products. However, such claims are largely extrapolated from studies involving engineered AuNPs and are not supported by a comprehensive physicochemical and toxicological evaluation of commercially available supplements. According to the exposure-based categorization proposed by Hanses et al., colloidal gold supplements belong to the group of products containing freely suspended nanoparticles, representing a high potential for direct biological interaction following ingestion [32]. Oral exposure is therefore of particular relevance. Unlike bulk gold, AuNPs can cross the intestinal barrier and enter systemic circulation [10,33]. Their absorption, biodistribution, persistence, and excretion are strongly influenced by particle size, shape, surface charge, aggregation state, and surface chemistry [33,34,35]. In general, smaller nanoparticles (<10 nm) exhibit higher absorption efficiencies and greater cellular interactions, whereas larger or aggregated particles are more likely to accumulate in reticuloendothelial tissues [33,34,35,36,37,38,39,40,41]. Depending on these characteristics, AuNPs may induce oxidative stress, alterations in gene expression, and other biological effects [41,42,43,44,45]. Despite their increasing commercial availability, colloidal gold supplements have not undergone safety evaluation under the EU Novel Food Regulation, and no acceptable daily intake (ADI) has been established by EFSA or other regulatory authorities [8,46,47]. Furthermore, gold nanoparticles intended for oral ingestion are not recognized as Generally Recognized as Safe (GRAS) by the U.S. Food and Drug Administration [48]. Consequently, many products are marketed without detailed nanoparticle characterization and often without information regarding particle size, colloidal stability, or long-term safety. Although numerous studies have investigated engineered AuNPs, considerably less information is available regarding commercially marketed colloidal gold supplements, particularly regarding the relationship between recommended daily intake levels and biological effects reported in experimental studies.

The aim of this study was to determine whether selected commercially available colloidal gold dietary supplements from the Romanian market meet the criteria for classification as nanomaterials. To achieve this, the products were characterized using complementary physicochemical techniques and compared with citrate-stabilized reference AuNPs. Estimated consumer exposure levels were subsequently contextualized using the human equivalent dose (HED) approach and available in vivo toxicological data. Preliminary biological assessment was performed using HCT-8 human colon adenocarcinoma cells, HEK293 human embryonic kidney cells, and three probiotic lactic acid bacteria strains (*Levilactobacillus brevis ZV1, Lactiplantibacillus plantarum SM4*, and *Limosilactobacillus reuteri LMG*).

The novelty of this study lies in combining the physicochemical characterization of commercially available colloidal gold supplements with an exposure-based evaluation that places manufacturer-recommended intake levels into the context of biological effects reported in experimental studies. Given that AuNP behavior is strongly influenced by particle characteristics and biological models, this approach does not establish safety thresholds but provides information relevant for the interpretation of potential long-term exposure.

## 2. Results

### 2.1. UV-VIS Spectroscopy Results

**Figure 1:**
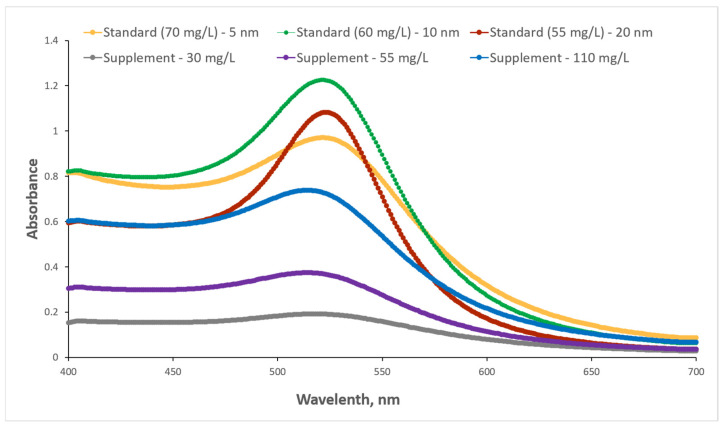
UV-VIS absorption spectra of colloidal gold dietary supplements and standard solutions.

UV-VIS spectroscopy was employed as a primary analytical technique to verify the presence of gold nanoparticles in the dietary supplements and to compare their optical signatures with those of standard colloidal gold nanoparticle solutions. The comparison with reference nanoparticle solutions was performed to qualitatively identify the characteristic surface plasmon resonance (LSPR) of gold nanoparticles, rather than to establish a direct size-dependent correlation. The UV-VIS absorption spectra are presented in Figure 1.

All three dietary supplements (110 mg/L, 55 mg/L, and 30 mg/L) exhibited well-defined LSPR bands, with absorption maxima located in the 514–519 nm range (Figure 1). This spectral signature is characteristic of metallic gold nanoparticles (Au^0^) and confirms that the investigated supplements contain gold in nanoparticulate colloidal form. The reference citrate-stabilized AuNP suspensions exhibited LSPR maxima in the 521–523 nm, showing the expected slight red-shift with increasing nominal particle size. The LSPR bands of the dietary supplements are consistently broader than those of the reference suspension. This spectral broadening indicates a polydisperse nanoparticle population, reflecting a wider size distribution and the presence of weak interparticle interactions in the supplement formulation, in contrast to the more monodisperse and electrostatically stabilized citrate-capped reference systems. Notably, the maximum absorbance values of the dietary supplements were significantly lower than those of the citrate-stabilized reference suspensions (Figure 1). This difference indicates a lower fraction of optically active, well-dispersed nanoparticles in the supplement formulations, consistent with their broader LSPR bands and polydisperse character, and reflects differences in dispersion state and colloidal organization between the dietary supplements and the reference systems.

### 2.2. Transmission Electron Microscopy (TEM) Results

**Figure 2:**
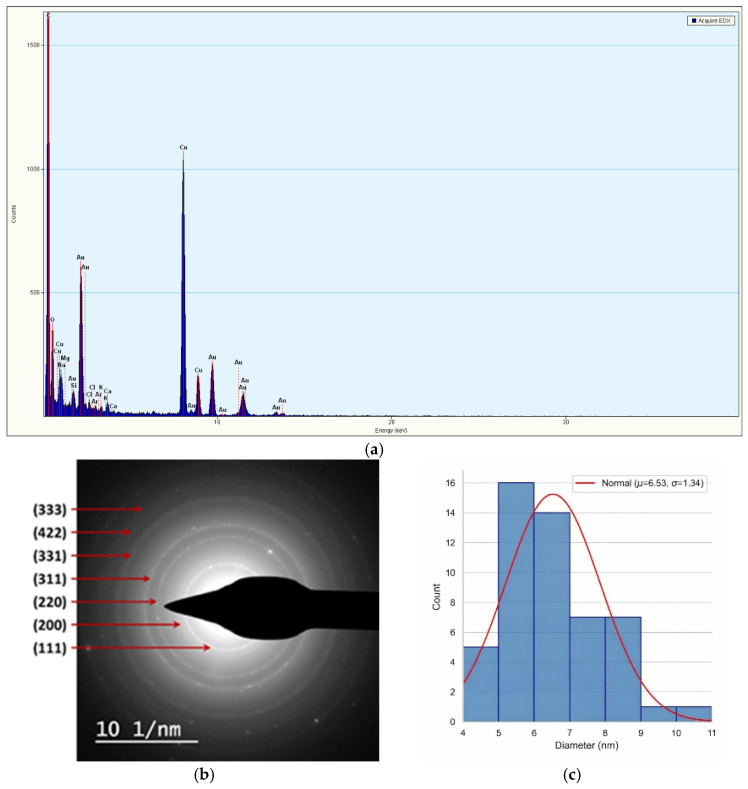

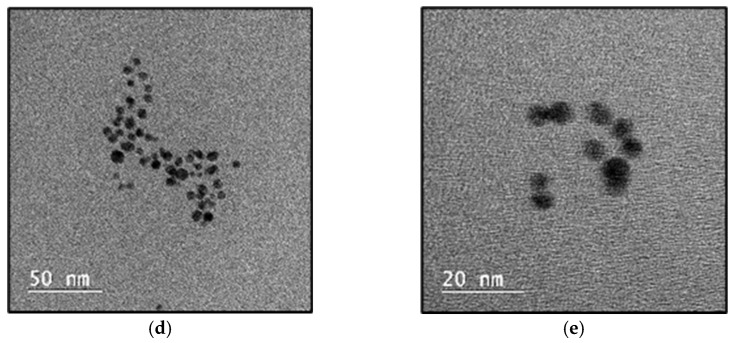
Representative BF-TEM micrographs, SAED pattern, and size distribution of the gold nanoparticles: (**a**) EDX spectrum; (**b**) the concentric diffraction rings; (**c**) size distribution histogram of the gold nanoparticles determined from TEM micrographs (*n* = 51); (**d**) Au NPs at 100,000×; (**e**) Au NPs at 280,000×.

Following the UV-VIS analysis, which confirmed the nanoparticulate nature of gold in the dietary supplements, transmission electron microscopy (TEM) was employed as a complementary technique to directly investigate nanoparticle morphology, size distribution, crystalline structure, and elemental composition at the nanoscale. Detailed TEM-based characterization was performed for the 55 mg/L dietary supplement (Figure 2).

Energy-dispersive X-ray spectroscopy (EDX) analysis confirms the elemental composition of the nanoparticles. The spectra are dominated by characteristic Au emission lines, confirming the presence of metallic gold as the primary constituent (Figure 2a). Strong Cu signals originate from the copper TEM grid, while C signals are attributed to the carbon support film. Minor O and trace elemental signals (e.g., Na, Mg, Si, Cl, Ca) are associated with surface adsorption, environmental contamination, and sample support, and do not indicate the presence of foreign metallic impurities in the nanoparticles.

Selected area electron diffraction (SAED) analysis reveals a series of well-defined concentric diffraction rings, indicating a polycrystalline nanoparticle ensemble. The diffraction rings are assigned to the (111), (200), (220), (311), (331), (422), and (333) crystallographic planes, confirming the presence of crystalline metallic gold (Au^0^) with Fm-3m symmetry (Figure 2b). No additional diffraction features associated with secondary crystalline phases, oxides, or salt-derived compounds are detected, indicating high structural purity of the nanoparticles. Quantitative ring-ratio analysis (di/d1) shows excellent agreement with reference values for face-centered cubic (fcc) gold, with deviations below 1.5% for all indexed reflections. Table 1 presents the comparison between the experimentally measured ring-ratio values and standard reference values for face-centered cubic (fcc), along with the corresponding Miller indices. Although the crystallographic data for gold are well established, the comparison is included to confirm the crystalline structure of the nanoparticles in the investigated commercial samples and to highlight the close agreement between experimental and reference values.

TEM micrographs recorded at 100,000× and 280,000× magnification reveal a population of well-defined gold nanoparticles with predominantly quasi-spherical morphology (Figure 2d,e). The nanoparticles are non-uniformly distributed on the carbon support film and exhibit localized agglomeration, appearing as small clusters and occasional chain-like assemblies. At higher magnification, individual primary nanoparticles are clearly resolved together with small multi-particle aggregates. No evidence of particle coalescence or fusion into larger nanostructures is observed, indicating that agglomeration occurs mainly through weak interparticle interactions rather than irreversible particle merging (Figure 2e). This behaviour is consistent with a colloidal system lacking a declared chemical stabilizing agent and dominated by weak electrostatic and capillary interactions during deposition and drying on the TEM grid.

Quantitative size analysis based on TEM image measurements shows a mean particle diameter of 6.53 nm with a standard deviation of 1.34 nm, indicating a moderately polydisperse nanoparticle population centered in the sub-10 nm size domain. The size distribution histogram exhibits a slight tail toward larger diameters, consistent with the presence of small aggregates in addition to primary nanoparticles (Figure 2c). No particles exceeding 11 nm were observed. This dimensional regime is particularly relevant from a biological perspective, as nanoparticles below 10 nm are associated with enhanced surface reactivity, increased bioavailability, and higher potential for cellular interactions [44,49,50,51,52].

### 2.3. Dynamic Light Scattering (DLS)

Dynamic light scattering (DLS) and zeta potential (ζ) measurements were employed to investigate the hydrodynamic size distributions, polydispersity, and surface charge properties of the colloidal gold dietary supplements.

**Figure 3:**
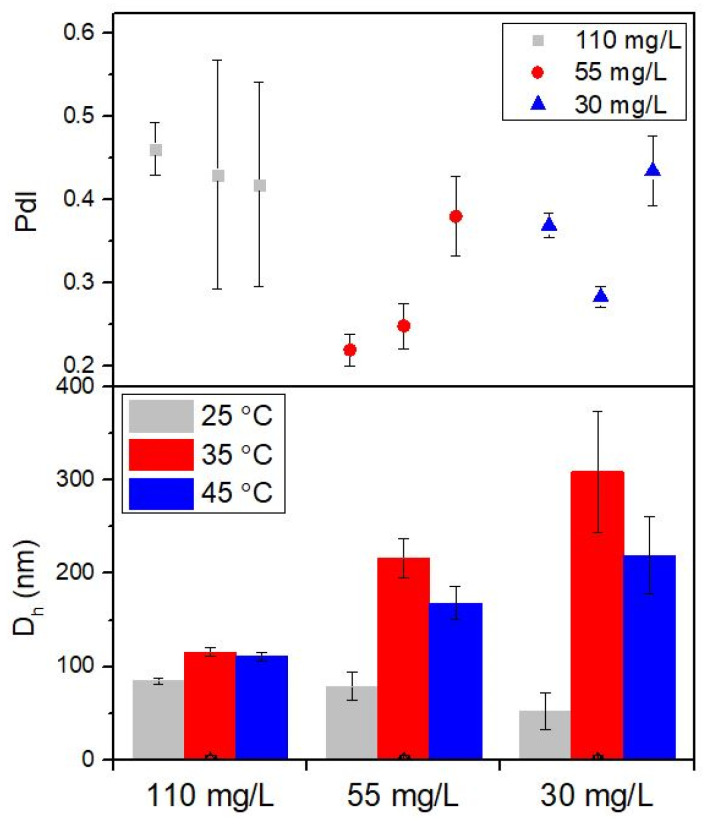
Hydrodynamic diameter (D_h_) and polydispersity index (PdI) of the main nanoparticle population from the colloidal gold supplements at different concentrations (110 mg/L, 55 mg/L, and 30 mg/L) and temperatures (25 °C, 35 °C, and 45 °C). Standard deviations as presented as error bars.

As observed in Figure 3, the 110 mg/L dispersions exhibit the smallest hydrodynamic diameters, with only minor variations across the investigated temperature range. A progressive increase in Dh is observed with decreasing concentration, with the largest hydrodynamic diameters corresponding to the 30 mg/L samples. For each concentration, increasing temperature from 25 °C to 45 °C is associated with an increase in the average hydrodynamic diameter, the effect being more pronounced at lower concentrations. The PdI values fall in the moderate-to-high range, indicating polydisperse systems rather than monodisperse nanoparticle populations. Higher PdI values are observed at lower concentrations and higher temperatures, corresponding to broader size distributions. The associated standard deviations increase for samples exhibiting larger Dh and higher PdI values, reflecting increased dispersion heterogeneity.

**Figure 4:**
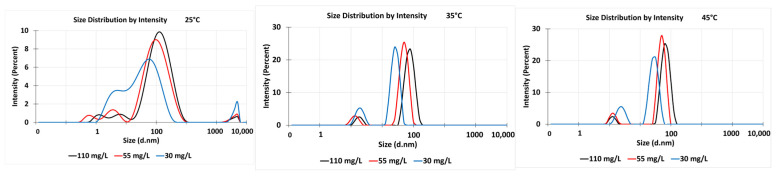
Intensity-weighted hydrodynamic size distributions of colloidal gold dietary supplements at concentrations of 110 mg/L, 55 mg/L, and 30 mg/L, measured by DLS at 25 °C, 35 °C, and 45 °C.

The intensity-weighted size distributions (Figure 4) reveal clear shifts in peak position and peak broadening with increasing temperature and decreasing concentration. For each concentration, increasing temperature leads to a displacement of the main intensity peak toward larger hydrodynamic diameters and to broadening of the distribution profile. These effects are weakest for the highest concentration (110 mg/L) and become increasingly pronounced for 55 mg/L and especially for 30 mg/L, where broader distributions and additional intensity contributions at larger sizes are observed. This behaviour indicates an enhanced contribution of larger particle populations and aggregates to the scattering signal at higher temperatures and lower concentrations.

**Figure 5:**
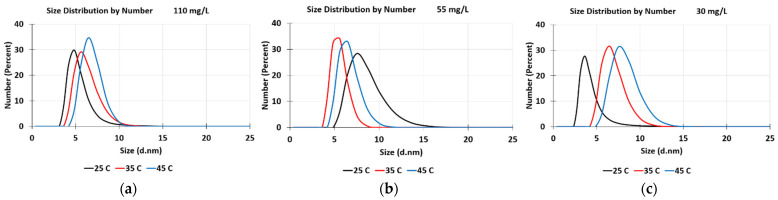
Number-weighted particle size distributions obtained by DLS for colloidal gold dietary supplements of (**a**) 110 mg/L, (**b**) 55 mg/L, and (**c**) 30 mg/L at three temperatures: 25 °C (black continuous line), 35 °C (red continuous line), and 45 °C (blue continuous line).

The number-weighted size distributions (Figure 5) show narrow, well-defined peaks in the sub-10 nm size range for all concentrations and temperatures. The peak positions exhibit only minor shifts with increasing temperature, and the distributions remain relatively narrow across all experimental conditions. Compared with the intensity-weighted distributions, the number-based distributions appear significantly more monodisperse, reflecting the reduced contribution of larger aggregates when particle size is weighted by particle count rather than scattering intensity. The combined analysis of intensity- and number-weighted size distributions highlights the intrinsic difference between scattering-dominated and number-based representations of particle size. While number-weighted distributions emphasize the dominant population of small primary nanoparticles, intensity-weighted distributions are strongly influenced by larger particles and aggregates due to the strong size dependence of light scattering intensity. The relatively high polydispersity indices observed for the dietary supplements indicate a heterogeneous nanoparticle population. However, the measured zeta potential values suggest good electrostatic stability, indicating that the colloidal systems remain stable despite their polydisperse nature. DLS measurements were not performed on the reference nanoparticle solutions, as these systems are well characterized, monodisperse, and their size distributions and polydispersity indices are provided by the manufacturer.

**Figure 6:**
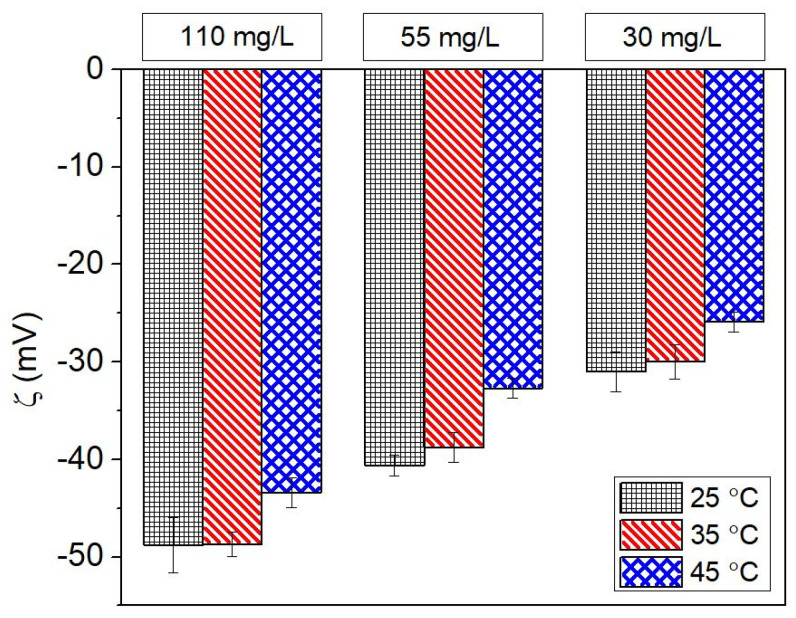
The average result of zeta potential with corresponding standard deviations presented as error bars of the colloidal gold supplements at different concentrations (110 mg/L, 55 mg/L, and 30 mg/L) and temperatures (25 °C, 35 °C, and 45 °C).

Figure 6 presents the zeta potential (ζ) values of the colloidal gold dietary supplements measured at three concentrations (110 mg/L, 55 mg/L, and 30 mg/L) and three temperatures (25 °C, 35 °C, and 45 °C), together with the corresponding standard deviations. All samples exhibit negative ζ-potential values across the investigated conditions, with magnitudes ranging approximately between −25 and −50 mV. Higher absolute values are observed at higher concentrations, while moderate temperature-dependent variations are evident for each concentration. The persistence of negative zeta potential values across all experimental conditions indicates a stable electrostatic surface charge environment for the nanoparticles and reflects a consistent interfacial chemistry of the colloidal system.

### 2.4. In Vitro Biological Evaluation

#### 2.4.1. Cytotoxicity Assay on HCT-8 Cells

**Figure 7:**
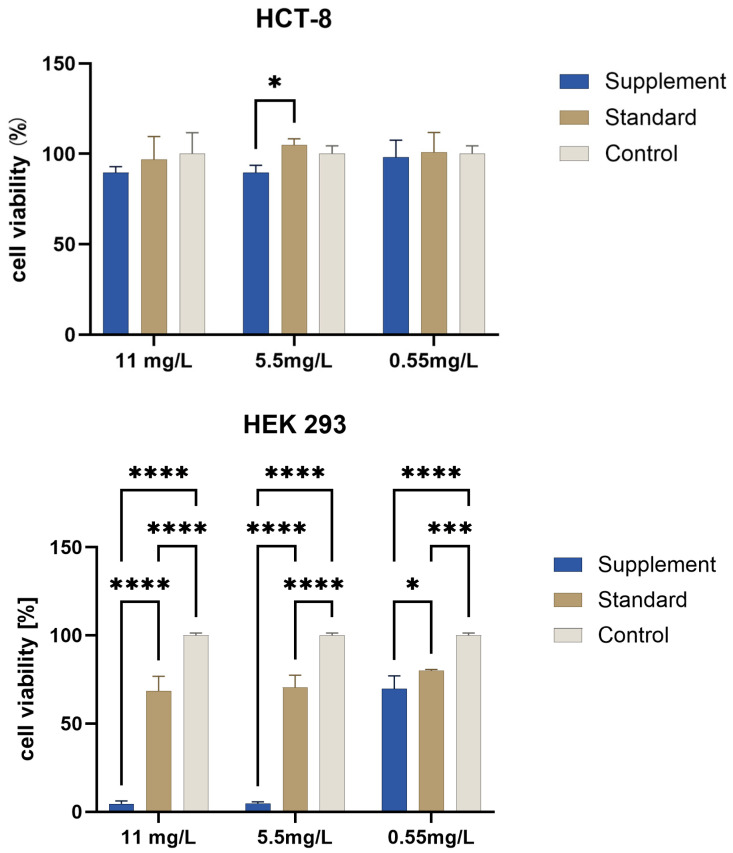
HCT-8/HEK 293 cell viability (%) after 24 h exposure to P2 (supplement-derived AuNPs) and P4 (citrate-stabilized standard AuNPs) at 11, 5.5, and 0.55 mg/L. Untreated cells were used as negative control (100%). Data are presented as mean ± SD (*n* = 4). Statistical analysis was performed using two-way ANOVA followed by Tukey’s multiple comparisons test (*p* < 0.05 (*), *p* < 0.001 (***), *p* < 0.0001 (****)).

All tested samples (P1–P6) showed viability values above 85%, indicating the absence of pronounced acute cytotoxicity under the experimental conditions. However, supplement-derived AuNPs generally induced slightly lower viability values compared with the reference nanoparticles. The most relevant difference was observed between samples P2 and P4. At 11 mg/L, cell viability was 90.5 ± 1.9% for P2 and 97.0 ± 12.4% for P4. At 5.5 mg/L, viability was 89.2 ± 4.0% for P2 compared with 104.8 ± 3.6% for P4, with a statistically significant difference detected by two-way ANOVA followed by Tukey’s multiple comparison test (*p* < 0.05). At 0.55 mg/L, no statistically significant differences were observed (Figure 7).

The consistently lower viability observed for supplement-derived AuNPs compared with standard nanoparticles indicates the presence of a modest but reproducible effect associated with the supplement formulations on the HCT-8 cell line. In HEK293 cells, the dietary supplement formulation exhibited pronounced cytotoxicity, reducing cell viability to 0% at concentrations of 11 mg/L and 5.5 mg/L. Even at the lowest tested concentration (0.55 mg/L), cell viability decreased by approximately 30%. In contrast, the standard formulation showed only moderate cytotoxic effects, resulting in a 25–30% reduction in cell viability at the lower concentrations tested.

#### 2.4.2. Effects of AuNP Samples on Lactic Acid Bacteria Growth

The effects of the AuNP samples investigated on the growth of three selected LAB strains (*L. brevis* ZV1, *L. plantarum* SM4, and *L. reuteri* LMG) were evaluated spectrophotometrically and by viable cell count (VCC).

**Figure 8:**
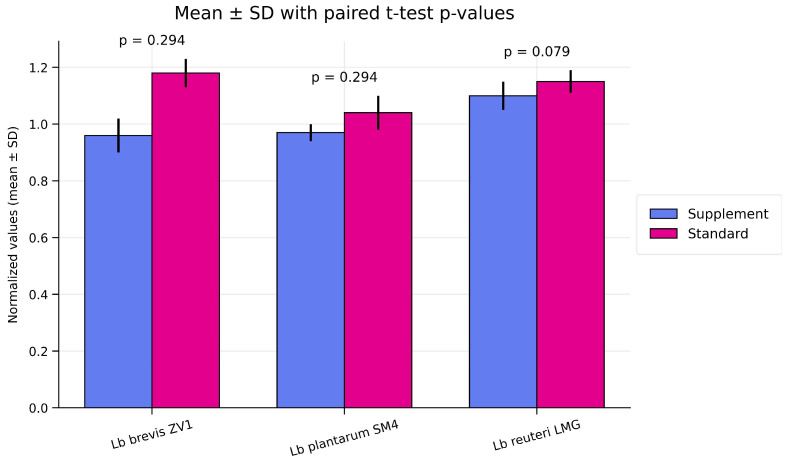
Graphical representation of the effect of P2 (supplement-derived AuNPs) and P4 (citrate-stabilized standard AuNPs) on probiotic strain growth normalized to control with *t*-test *p*-values (*p* < 0.05, *p* < 0.01, *p* < 0.001, *p* < 0.0001).

**Figure 9:**
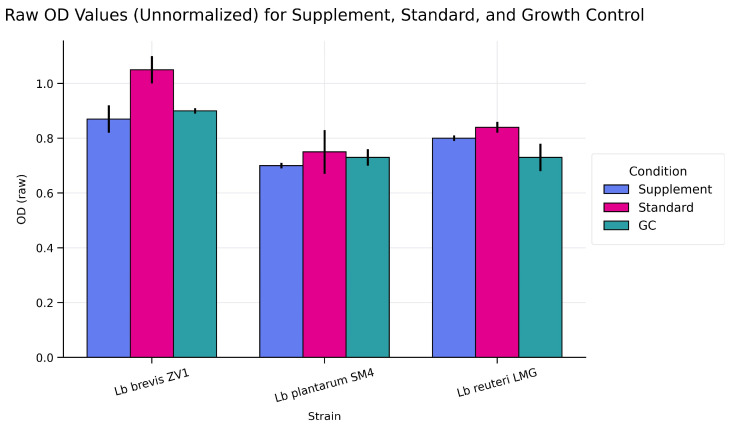
Comparison of Raw OD (540 nm) values for P2 (supplement-derived AuNPs) and P4 (citrate-stabilized standard AuNPs), and growth control (GC) in three *Lactobacillus Strains*.

The statistical comparison between supplement-derived AuNPs_norm and citrate-stabilized reference AuNPs_norm samples for each *Lactobacillus* strain shows different patterns of response (Figure 8 and Figure 9).

For *Levilactobacillus* (Lb) *brevis* ZV1 strain (isolated from sauerkraut brine), the difference between supplement-derived AuNPs_norm and citrate-stabilized reference AuNPs_norm is statistically significant (*p* = 0.015). The mean normalized value for supplement-derived AuNPs is approximately 0.96, while the mean for citrate-stabilized reference AuNPs is about 1.17. This indicates that citrate-stabilized reference AuNPs treatment significantly increases growth compared with supplement-derived AuNPs. Biologically, this suggests that citrate-stabilized reference AuNPs enhance growth or metabolic activity in this strain, producing a stimulatory effect above the growth control baseline.

For *Lactiplantibacillus* (Lb) *plantarum* SM4 strain (isolated from sour cream), the paired t-test shows no significant difference between supplement-derived AuNPs_norm and citrate-stabilized reference AuNPs_norm, with *p* = 0.367. Although the mean values differ slightly, the variability between replicates is too large to support a significant effect. This indicates that SM4 is relatively unaffected by either supplement-derived AuNPs or citrate-stabilized reference AuNPs and maintains stable growth across treatments. The strain may have more robust regulatory or resistance mechanisms that minimize treatment effects.

For *Limosilactobacillus* (Lb) *reuteri* LMG (isolated from a commercial probiotic product), the *p*-value of 0.090 indicates a trend toward significance, but it does not reach the conventional threshold of 0.05. Both supplement-derived AuNPs_norm (approximately 1.10) and citrate-stabilized reference AuNPs_norm (approximately 1.15) show growth stimulation compared with the control, but the sample size (three replicates) limits statistical power. Biologically, this strain appears to respond positively to both treatments, but further replicates would be needed to confirm whether the difference is meaningful.

**Figure 10:**
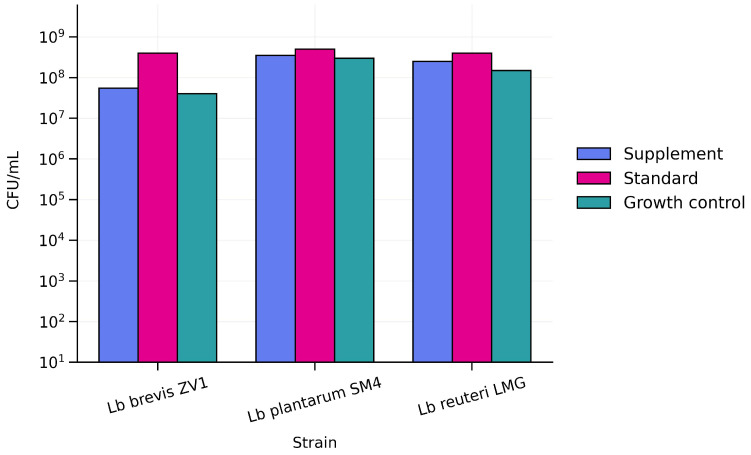
Growth Response of LAB Strains to *P2* (supplement-derived AuNPs) and P4 (citrate-stabilized standard AuNPs) treatments (CFU/mL, log scale).

Exposure to supplement-derived AuNPs and citrate-stabilized reference AuNPs produced distinct effects on the viable cell counts (VCC) of the tested LAB strains (Figure 10). For *Lb. brevis* ZV1, supplement-derived AuNPs treatment resulted in approximately 1 × 10^8^ CFU/mL, like the control (~1 × 10^8^ CFU/mL), whereas citrate-stabilized reference AuNPs increased growth to approximately 1 × 10^9^ CFU/mL. *Lb. plantarum* SM4 exhibited high viability across all conditions, with supplement-derived AuNPs and citrate-stabilized reference AuNPs both yielding ~1 × 10^9^ to 2 × 10^9^ CFU/mL, compared with ~8 × 10^8^ to 1 × 10^9^ CFU/mL in the control. A similar pattern was observed for *Lb. reuteri* LMG, where supplement-derived AuNPs and citrate-stabilized reference AuNPs maintained or slightly enhanced viable counts (~1 × 10^9^ to 1.5 × 10^9^ CFU/mL) relative to the control (~7 × 10^8^ to 1 × 10^9^ CFU/mL). Overall, citrate-stabilized reference AuNPs consistently resulted in equal or higher CFU/mL values compared with both supplement-derived AuNPs-treated and control cultures, suggesting a potential stimulatory effect on bacterial growth for the tested LAB strains.

## 3. Discussions

### 3.1. Physicochemical Characterization of Gold Colloidal Dietary Supplements

The physicochemical characterization performed in this study demonstrated that the investigated dietary supplements contain gold in colloidal nanoparticulate form. UV-VIS spectroscopy revealed the characteristic localized surface plasmon resonance of gold nanoparticles, indicating the presence of dispersed AuNPs. TEM analysis confirmed predominantly spherical nanoparticles with diameters ranging from 4 to 11 nm. These results are supported by the number-weighted DLS distributions, which showed dominant nanoparticle populations within the same range. To evaluate colloidal stability under conditions relevant both to product storage and potential biological exposure, DLS and zeta potential measurements were performed at different temperatures. Although variations in hydrodynamic diameter were observed, the consistently negative zeta potential values, maintained even at 35 °C and 45 °C, indicate effective electrostatic stabilization of the dispersions.

### 3.2. Estimated Daily Intake from the Investigated Dietary Supplements

The estimated daily intake of gold from dietary supplements investigated in the present study was derived from the gold concentrations reported on the product labels and the manufacturer-specified intake instructions. The analyzed products consist of distilled water containing colloidal gold nanoparticles with reported particle sizes in the range of approximately 0.5–10 nm and are intended for internal use. According to the label information, the recommended intake is expressed as 5–15 mL per administration, taken one to four times per day, resulting in a broad interval of possible daily consumption volumes. To contextualize potential intake levels, daily gold exposure was estimated for a reference adult body weight of 70 kg, commonly used in exposure and toxicological assessments. In addition, calculations were performed for a body weight of 50 kg to illustrate how lower body mass may influence exposure when expressed on a per-kilogram basis. These estimates provide a quantitative framework for defining the range of possible daily gold intake associated with the investigated supplements and serve as a basis for comparison with experimental exposure levels discussed in the following section.

The estimated daily intake of gold (mg/day) was calculated using Formula (1):

daily intake (mg/day) = concentration (mg/mL) × volume per dose (mL) × frequency (dose/day),(1)

To assess potential health risks, the daily intake was normalized to body weight (mg/kg/day) (Formula (2)).

daily intake per body weight (mg/kg/day) = daily intake (mg/day) ÷ body weight,(2)

Table 2 presents the estimated daily gold intake associated with investigated dietary supplements, expressed as mg/day and mg/kg/day for individuals weighing 50 and 70 kg.

### 3.3. Evidence from In Vivo Studies

Gold nanoparticles have been extensively investigated in vivo experimental systems, and a substantial body of literature has documented their biological effects across a wide range of exposure conditions [33,35,53,54]. The literature discussed below is used to contextualize experimentally reported dose ranges and observed effects in relation to the oral exposure scenario.

Zhang et al. investigated the biological effects of smaller gold nanoparticles (13.5 nm) administered via intravenous, oral, and intraperitoneal routes in mouse models under repeated dosing conditions [33]. Biological endpoints were evaluated after 14 and 28 days of exposure. Clear differences emerged between administration routes. While intravenous and intraperitoneal administration primarily resulted in direct systemic exposure, repeated oral administration was associated with more pronounced alterations in immune-related parameters. Specifically, oral exposure led to significant changes in spleen index and hematological parameters, including an increase in spleen index and a reduction in red blood cell counts. These effects were detectable after 14 days of exposure and became more pronounced after 28 days, demonstrating a clear time- and dose-dependent progression. In the same context of repeated oral exposure, Evariste et al. evaluated the biological effects of food-grade gold (E175) in rodent models [54]. In this work, animals were exposed daily to E175 for a continuous period of 90 days, allowing assessment of subchronic biological responses under conditions that approximate long-term dietary intake. E175, which consisted predominantly of larger gold particles in the submicron-to-nanoscale range, was administered via the oral route throughout the entire exposure period. Although no overt systemic toxicity or major histopathological alterations were observed, changes in gut microbiota composition and local intestinal immune homeostasis were detected. Female animals exhibited an increased Firmicutes/Bacteroidetes ratio and higher Proteobacteria abundance, while a reduction in short-chain fatty acid production was observed in both sexes. These alterations were accompanied by evidence of low-grade intestinal inflammation, particularly in females, identifying the gastrointestinal tract as a sensitive target of long-term oral exposure. Comparable results were reported in a subchronic oral toxicity study by Sun et al., who administered citrate-coated spherical gold nanoparticles with a mean diameter of 53 nm to male and female mice via daily oral dosing for 90 days [35]. While no mortality or pronounced clinical toxicity was observed, minor dose-dependent alterations in selected histopathological and hematological parameters were detected at the highest exposure levels. The authors explicitly discussed their results in relation to those by Zhang et al. [33], attributing differences in observed biological effects primarily to variations in nanoparticle size, surface properties, and exposure paradigms. This body of evidence underscores the importance of considering route-, size-, and time-dependent factors when evaluating the biological relevance of gold nanoparticles intended for oral consumption.

### 3.4. Comparison with Experimental Exposure Levels

The intake ranges estimated for the investigated supplements (Section 3.2) were compared with exposure levels reported in the experimental studies discussed in Section 3.3. To enable cross-species correspondence between animal dosing regimens and potential human exposure, reported animal doses were converted to human equivalent doses (HED) using body surface area scaling, which is widely applied in toxicological risk assessment [55]. In this approach, HED values (mg/kg) are derived from animal dose (mg/kg) multiplied by the ratio of species-specific conversion factors (K_m_) (Formula (3)). We used body surface area scaling (mouse-to-human factor 3/37).

HED (mg/kg) = Animal dose (mg/kg) × (K_m_ animal/K_m_ human), (3)

The studies selected for HED comparison are summarized in Table 3.

The comparison indicates that the estimated intake levels may approach, overlap with, or exceed exposure levels reported to induce measurable biological responses in experimental models. The available evidence further suggests that biological effects may occur in the absence of overt systemic toxicity and are influenced not only by administered dose but also by nanoparticle size, morphology, surface characteristics, and exposure duration. However, the exposure estimates presented here are based on manufacturer-recommended intake levels and product information and should therefore be considered indicative rather than representative of actual consumer exposure. Consequently, the HED-based comparison provides a framework for contextualizing potential exposure and supports the need for nanospecific assessment of colloidal gold formulations.

### 3.5. In Vitro Biological Evaluation of Gold Nanoparticle Formulations

#### 3.5.1. Cytotoxic Effects of Supplement-Derived AuNPs

The present study demonstrates that supplement-derived AuNPs are not biologically inert, as modest but reproducible reductions in HCT-8 cell viability were observed following 24 h of exposure. Although no pronounced acute cytotoxicity was detected and viability values remained above 85% in all cases, supplement-derived samples consistently exhibited lower viability compared with citrate-stabilized reference AuNPs. The most relevant comparison was observed between P2 and P4, evaluated at identical nominal gold concentrations. Under these conditions, P2 induced significantly lower viability at 5.5 mg/L, suggesting that the cellular response is influenced primarily by formulation-dependent physicochemical properties rather than by total gold concentration alone. The viability value observed for P4 (104.8 ± 3.6%) was considered comparable to the untreated control and does not indicate a biologically meaningful proliferative response.

A more pronounced response was observed in the HEK293 cell line. The dietary supplement formulation exhibited substantially higher cytotoxicity than the corresponding standard AuNP formulation, resulting in complete loss of cell viability at concentrations of 11 mg/L and 5.5 mg/L. Notably, a reduction of approximately 30% in viability was still evident at the lowest concentration tested (0.55 mg/L). In contrast, the standard formulation induced only moderate effects on cell viability. These findings suggest that specific physicochemical features of the commercial formulation, including particle surface properties, aggregation behavior, or the presence of formulation-related constituents, may contribute to the enhanced cytotoxic response observed in HEK293 cells.

Taken together, the results do not provide evidence for a significant antitumoral activity of the investigated colloidal gold supplements. Despite claims occasionally encountered in non-scientific sources regarding potential anticancer effects, the observed responses in HCT-8 cells do not indicate a substantial inhibition of tumor cell viability under the short-term exposure conditions employed in this study. Consequently, the tested formulations cannot be considered antitumoral agents based on the present in vitro data. At the same time, the measurable cytotoxic effects observed, particularly in HEK293 cells, raise important questions regarding the biological consequences of prolonged exposure. Given that colloidal gold supplements are often consumed repeatedly over extended periods, chronic exposure to small-sized nanoparticles may result in cumulative cellular effects that cannot be captured by short-term assays. Therefore, further investigations involving repeated-dose exposure protocols, longer incubation periods, and mechanistic studies are warranted to better understand the potential health implications associated with long-term consumption of colloidal gold dietary supplements.

#### 3.5.2. Effects on Lactic Acid Bacteria

The interaction between investigated samples and probiotic lactic acid bacteria revealed a biological profile distinct from that observed in tumor cells. Across all tested strains (*Levilactobacillus brevis* ZV1, *Lactiplantibacillus plantarum* SM4, and *Limosilactobacillus reuteri* LMG), neither supplement-derived AuNPs nor citrate-stabilized reference AuNPs samples inhibited bacterial growth after short-term exposure. Normalized growth values were comparable to or slightly higher than those of untreated controls, indicating the absence of antimicrobial activity at the tested concentration. In some cases, a strain-dependent increase in growth was observed, particularly for *L. reuteri*, suggesting that the investigated nanoparticles may interact with bacterial systems in a manner that does not impair proliferation and may even promote growth under specific conditions. From a short-term perspective, these findings suggest that the tested formulations are unlikely to disrupt beneficial LAB populations. However, the present evaluation was limited to acute exposure conditions. While no inhibitory effects were detected within the investigated timeframe, the potential impact of prolonged or repeated exposure remains unclear. Long-term studies are therefore required to fully elucidate whether chronic exposure could alter bacterial physiology, metabolic activity, or community balance.

## 4. Materials and Methods

### 4.1. Sample Description

Three commercial colloidal gold dietary supplements with declared gold concentrations of 30, 55, and 110 mg/L were investigated. The products were manufactured in Romania and acquired from the local market. According to the product labels, all supplements consisted exclusively of colloidal gold dispersed in distilled water and were described as electro-colloidal formulations suggesting stabilization primarily through electrostatic mechanisms, with no stabilizing agent specified on the product label. These formulations are similar across the investigated samples, differing primarily in their declared gold concentration. For comparison, well-characterized reference gold nanoparticle suspensions with nominal particle diameters of 5, 10, and 20 nm were used as benchmark materials for physicochemical and biological characterization. According to the technical datasheets provided by the manufacturer, these reference materials contained citrate-stabilized gold nanoparticles dispersed in distilled water. The manufacturer-reported concentrations of the reference suspensions were expressed as particle number densities, namely 5.5 × 10^13^, 6.0 × 10^12^, and 6.54 × 10^11^ particles/mL for the 5, 10, and 20 nm nanoparticles, respectively. These values were transformed into estimated mass concentrations assuming spherical nanoparticle geometry, as supported by the TEM-based morphological analysis provided by the manufacturer, and using the bulk density of gold for the mass calculations, yielding approximate concentrations of 70, 60, and 55 mg/L. The gravimetric calculation was performed according to the approach described by Shang and Gao, following the particle mass calculation commonly used for nanoparticle suspensions [56,57]. To further verify the estimated gold mass concentration obtained by the gravimetric approach, the 20 nm citrate-stabilized reference AuNP suspension was additionally evaluated by UV-VIS spectroscopy (Shimadzu Corporation, Kyoto, Japan). The gold concentration was independently estimated from the absorbance at 400 nm using the method proposed by Khlebtsov et al. [58]. The UV-VIS-derived concentration was in close agreement with the gravimetric estimate, supporting the validity of the nominal concentration used in this study. The reference gold nanoparticle solutions were supplied by Sigma-Aldrich.

### 4.2. Analytical Methods

#### 4.2.1. UV-VIS Spectroscopy

In this study, UV-VIS spectra were recorded using a Shimadzu UV-1900i spectrophotometer over the 400–700 nm wavelength range. Measurements were performed in quartz cuvettes with a 1 cm optical path length, using distilled water as the reference in the reference beam. Spectra were acquired in absorbance mode under the instrument’s standard operating conditions. No dilution was performed for any of the samples, including both commercial colloidal gold dietary supplements and reference gold nanoparticle suspensions. The recorded absorbance values were close to unity and remained within the linear response range of the spectrophotometer, allowing direct comparison between samples.

#### 4.2.2. Transmission Electron Microscopy (TEM)

For TEM analysis, 10 µL of each colloidal suspension was deposited onto carbon-coated 300 mesh copper grids (Ted Pella Inc., Redding, CA, USA). Excess liquid was gently removed using fine filter paper, and the grids were allowed to dry under ambient conditions prior to analysis. TEM observations were performed using a Tecnai F20 G2 TWIN transmission electron microscope (Thermo Fisher Scientific, Waltham, MA, USA), operated in bright-field (BF-TEM) mode at an accelerating voltage of 200 kV. The instrument was equipped with selected area electron diffraction (SAED) and energy-dispersive X-ray spectroscopy (EDX) detectors. Image acquisition was performed using DigitalMicrograph software (version 2.12, Gatan Inc., Pleasanton, CA, USA), and image processing and analysis were carried out using TEM Imaging & Analysis (TIA) software (version 4.6.4).

SAED patterns were acquired to assess the crystalline nature of the gold nanoparticles. X-ray diffraction (XRD) analysis was attempted. However, due to the low concentration of nanoparticles in the investigated colloidal solutions, no reliable diffraction signal could be obtained. EDX analysis was employed to confirm the presence of gold and to detect any additional elements potentially associated with stabilizing agents or impurities.

#### 4.2.3. Dynamic Light Scattering (DLS) and Zeta Potential

Dynamic Light Scattering (DLS) measurements were conducted using a Malvern ZetaSizer Nano-ZS instrument (Malvern Instruments, Malvern, UK). The sample formulations were equilibrated prior to the measurements, and the records were performed with 10 runs at temperatures of 25 °C, 35 °C, and 45 °C in order to explore temperature-dependent effects under biologically relevant conditions. This approach allows the assessment of the sensitivity of the colloidal systems to environmental changes, which may influence their stability during storage and potential biological exposure. In the measurement’s settings, the properties of double-distilled water used in solution preparations, standard viscosity, refractive index, and dielectric constant at the corresponding temperature were considered. DLS size distributions were acquired primarily as intensity-weighted distributions, as provided by the instrument software, and were further converted into number-weighted distributions for comparative analysis.

### 4.3. In Vitro Biological Assays

To provide a preliminary assessment of the biological interactions of supplement-derived AuNPs following oral exposure, biological evaluation was conducted using HCT-8 human colon adenocarcinoma cells, HEK293 human embryonic kidney cells, and selected probiotic lactic acid bacteria strains.

HCT-8 cells were chosen in view of the antitumoral claims frequently associated with colloidal gold products, whereas *Levilactobacillus* (Lb) *brevis* ZV1, *Lactiplantibacillus* (Lb) *plantarum* SM4, and *Limosilactobacillus* (Lb) *reuteri* LMG were selected as representative beneficial microorganisms of the gut microbiota potentially exposed after oral ingestion.

Although HEK293 cells do not fully reproduce the complexity of renal tissue in vivo, they are widely used as a human cell model for preliminary toxicological screening and biocompatibility studies involving nanomaterials.

#### 4.3.1. Cell Viability Assay on HCT-8 Cells and HEK293

HCT-8 and HEK293 were cultured in Dulbecco’s Modified Eagle Medium (DMEM; Sigma-Aldrich, St. Louis, MO, USA) supplemented with 10% fetal bovine serum (FBS) and 1% penicillin-streptomycin. Cells were maintained at 37 °C in a humidified atmosphere containing 5% CO_2_.

Six gold nanoparticle (AuNP) solutions described in 4.1 were used for the in vitro experiments. Three samples corresponded to commercial colloidal gold supplements (P1–P3), with declared gold concentrations of 30 mg/L (P1), 55 mg/L (P2), and 110 mg/L (P3), respectively. The reference systems consisted of three citrate-stabilized AuNP solutions (P4–P6), corresponding to nanoparticles with nominal diameters of 20 nm (P4), 10 nm (P5), and 5 nm (P6), with estimated mass concentrations of approximately 55, 60, and 70 mg/L. For each investigated sample (P1–P6), serial dilutions were prepared in phosphate-buffered saline (PBS) to obtain dilution ratios of 1:5, 1:10, and 1:100 relative to the corresponding stock solution. All supplements and reference AuNP solutions were therefore tested under identical dilution conditions. The diluted suspensions were added to the cell culture medium, and cells were exposed to the tested solutions for 24 h under standard incubation conditions.

Cell viability was evaluated using MTT (3-(4,5-dimethylthiazol-2-yl)-2,5-diphenyltetrazolium bromide) reduction assay. Following 24 h exposure to the diluted AuNP samples (P1–P6), the culture medium was removed and replaced with MTT solution (100 µg/mL). Cells were incubated for 4 h at 37 °C in a humidified atmosphere containing 5% CO_2_ to allow the formation of formazan crystals. After incubation, the MTT solution was discarded, and the resulting formazan crystals were dissolved in 100 µL dimethyl sulfoxide (DMSO). Absorbance was measured at 540 nm using a Synergy HTX Multi-Mode Microplate Reader (Biotek, Winooski, VT, USA). Cell viability was expressed as percentage relative to untreated control cells (negative control, 100%). All experiments were performed in quadruplicate (*n* = 4), and the results are shown as means ± standard deviation. To assess statistical significance, a 2-way ANOVA was performed using GraphPad Prism Software version 10.45.4.73 (Boston, MA, USA). Following this, Tukey’s test was used to determine significant differences between the means, with a significance threshold set at *p* < 0.05.

#### 4.3.2. Lactic Acid Bacteria Growth Assay

- *Levilactobacillus* (Lb) *brevis* ZV1 strain (isolated from sauerkraut brine);
- *Lactiplantibacillus* (Lb) *plantarum* SM4 strain (isolated from sour cream);
- *Limosilactobacillus* (Lb) *reuteri* LMG (isolated from a commercial probiotic product).

Each of the 3 LAB cultures was refreshed in MRS broth medium and subsequently centrifuged at 6000 rpm for 10 min. The supernatant was discarded, and a portion of the resulting pellet was resuspended in distilled water to prepare a bacterial suspension adjusted to a turbidity equivalent to 0.5 McFarland standard. These suspensions were used for the inoculation of a 24-well plate to establish the effect of supplement-derived AuNPs and citrate-stabilized reference AuNPs samples on LAB growth, after 24 h of incubation, at 37 °C, in anaerobic conditions. Each gold sample was tested at final concentration of 27.5 ppm, in MRS medium (in triplicate for each LAB strain). Also, in all working variants, the wells were inoculated with LAB suspension at final concentration of 1.5 × 10^6^ CFU/mL, including the growth control.

The evaluation of stimulatory effect toward LAB growth was performed using a spectrophotometric method and the VCC (viable cell count) method. The qualitative evaluation was performed by spectrophotometric determination of the intensity of the LAB cultures, measuring the absorption at 540 nm, using an ELISA reader—model SYNERGY HTX multi-mode reader (Agilent BioTek, Winooski, VT, USA). Growth data for each probiotic strain exposed to supplement-derived AuNPs and citrate-stabilized reference AuNPs were normalized to their respective untreated controls. Normalized growth was calculated as the ratio between the optical density (OD) of treated samples and the mean OD of control samples at the corresponding time point and expressed as relative growth (fold change). For visualization, normalized growth values were plotted as mean ± standard deviation (SD) from at least three independent experiments. Each treatment condition (supplement-derived AuNPs and citrate-stabilized reference AuNPs) was compared against the control using a two-tailed unpaired Student’s *t*-test, after confirming data normality. The resulting *p*-values were included directly on the graphs to indicate statistical significance. Statistical analysis and plotting were performed using GraphPad Prism Software.

The effect of supplement-derived AuNPs and citrate-stabilized reference AuNPs on the growth of probiotic lactic acid bacteria (LAB) strains was assessed by quantifying viable cell counts (CFU/mL). Three strains, *Levilactobacillus brevis* ZV1, *Lactiplantibacillus plantarum* SM4, and *Limosilactobacillus reuteri* LMG, were cultured under standard conditions and exposed to supplement-derived AuNPs and citrate-stabilized reference AuNPs for the defined incubation period; untreated cultures served as growth controls. After incubation, samples were serially diluted, plated on appropriate selective media, and incubated for colony enumeration. Resulting CFU/mL values were plotted on a logarithmic scale to compare the impact of each treatment relative to the untreated control. Data were interpreted by examining differences in viable counts among supplement-derived AuNPs, citrate-stabilized reference AuNPs, and control-treated cultures.

## 5. Conclusions

The colloidal gold dietary supplements investigated were shown to meet the criteria of engineered nanomaterials based on their physicochemical characteristics. Transmission electron microscopy revealed a relatively narrow particle size distribution, while SAED, EDX, and UV-VIS analysis confirmed the presence of metallic gold nanoparticles. In accordance with the 2022 European Union definition of nanomaterials, the number-based size distribution supports the classification of these products as nanomaterial-containing formulations. The in vitro biological evaluation demonstrated that supplement-derived AuNPs do not exert significant cytotoxic or antitumoral effects on HCT-8 cells under the investigated conditions. In contrast, the dietary supplement formulation exhibited markedly higher cytotoxicity toward HEK293 cells than the standard AuNP formulation, indicating that its biological effects are cell type-dependent and influenced by formulation-specific characteristics. In addition, no inhibitory effects were detected on selected lactic acid bacteria strains under short-term exposure conditions.

The exposure contextualization approach provided additional perspective on the potential relevance of long-term oral intake. Although the investigated products did not exhibit antitumoral activity, the observed differences between supplement-derived and reference nanoparticles indicate that colloidal gold supplements should not be regarded as biologically inert.

Overall, the findings support the need to evaluate colloidal gold supplements within a regulatory and toxicological framework applicable to nanomaterials. At a minimum, products containing gold nan##oparticles should undergo characterization of particle size distribution, concentration, surface coating, and colloidal stability, complemented by appropriate long-term biological evaluation prior to commercialization.

## Figures and Tables

**Table 1:** The comparison between the experimentally measured ring-ratio values and standard reference values for face-centered cubic (fcc), along with the corresponding Miller indices.

No.	Au fcc Ring-Ratio Values (di/d1)		Miller Indices
	Measured	Standard	
I = 1	1.000	1.000	(111)
2	0.868	0.866	(200)
3	0.618	0.612	(220)
4	0.524	0.522	(311)
5	0.397	0.397	(331)
6	0.359	0.354	(422)
7	0.337	0.333	(333)

**Table 2:** Daily gold intake from colloidal gold dietary supplements marketed in Romania.

Supplement	Concentration (mg/mL)	Particle Size (nm)	Single Dose (mL)	Daily Frequency	Daily Intake (mg/day)	Daily Intake per 50 kg (mg/kg/day)	Daily Intake per 70 kg (mg/kg/day)
30 mg/L	0.030	0.5–10	5–15	1–4	0.15–1.8	0.003–0.036	0.0021–0.0257
55 mg/L	0.055	0.5–10	5–15	1–4	0.275–3.3	0.0055–0.066	0.0039–0.0471
110 mg/L	0.110	0.5–10	5–15	1–4	0.55–6.6	0.01–0.132	0.0079–0.0943

**Table 3:** Comparison of estimated oral exposure with experimental exposure levels used in the present study.

Reference	Experimental Model	Nanomaterial Characteristics (Size, Shape, Surface Coating)	Exposure Duration	Animal Dose (mg/kg/day)	HED (mg/kg)	Biological Effects	Relationship to Estimated Intake Levels *
Zhang et al. [33]	Mice	13.5 nm, spherical, citrate AuNPs	14–28 days	0.55	0.045	Increased spleen index and reduced red blood cell count, indicating effects on immune-related and hematopoietic endpoints	Upper estimated intake levels approach this HED value
Zhang et al. [33]	Mice	13.5 nm, spherical, citrate AuNPs	14–28 days	1.10	0.089	More pronounced increase in spleen index, alterations in hematological parameters, and decreased body weight	Maximum estimated intake levels may approach or exceed this HED value
Sun et al. [35]	Mice	53 nm, spherical, citrate AuNPs	90 days	20	1.6	No pronounced hematological or histopathological alterations following repeated exposure	Estimated intake levels remain substantially below this HED value
Evariste et al. [54]	Mice	Food-grade gold (E175) containing~28% nanoplates, 60–100 nm	90 days	0.01	0.0008	Increased Firmicutes/Bacteroidetes ratio and Proteobacteria abundance in females, reduced short-chain fatty acid production in both sexes, evidence of low-grade intestinal inflammation, and altered intestinal cytokine profiles	Estimated intake levels overlap with and exceed this HED value

* Estimated intake ranges for the investigated supplements were 0.0021–0.0943 mg/kg/day for a 70 kg adult and 0.003–0.132 mg/kg/day for a 50 kg adult (Table 2).

## Data Availability

The original contributions presented in this study are included in the article. Further inquiries can be directed to the corresponding author(s).

## Abbreviations

The following abbreviations are used in this manuscript:

ISO	International Organization for Standardization
OECD	Organization for Economic Cooperation and Development
TEM	Transmission Electron Microscopy
SAED	Selected Area Electron Diffraction
EDX	Energy Dispersive X-ray Spectroscopy
LSPR	Localized Surface Plasmon Resonance
WHO	World Health Organization
EFSA	European Food Safety Authority
FAO	Food and Agriculture Organization
ECHA	European Chemicals Agency
DNA	deoxyribonucleic acid
ADI	Acceptable Daily Intake
GRAS	Generally Recognized as Safe
DLS	Dynamic Light Scattering
FBS	Fetal Bovine Serum
LAB	Lactic Acid Bacteria
OD	Optical Density
CFU	Colony Forming Units
VCC	Viable Cell Count
fcc	face-centered cubic
HED	Human Equivalent Doses
